# The risk of cardiovascular complications in inflammatory bowel disease

**DOI:** 10.1007/s10238-020-00639-y

**Published:** 2020-08-12

**Authors:** Piotr Czubkowski, Marcin Osiecki, Edyta Szymańska, Jarosław Kierkuś

**Affiliations:** Department of Gastroenterology, Hepatology, Nutritional Disorders and Pediatrics, The Childrens’ Memorial Health Institute, Warsaw, Poland

**Keywords:** Inflammatory bowel disease, Endothelial dysfunction, Ischemic heart disease, Atherosclerosis

## Abstract

Inflammatory bowel disease (IBD) is a chronic, relapsing disease of unknown etiology involving gastrointestinal tract. IBD comprises two main entities: ulcerative colitis and Crohn’s disease. Several studies showed increased risk of cardiovascular complications in chronic inflammatory disorders, especially during IBD relapses. Endothelium plays a role in physiologic regulation of vascular tone, cell adhesion, migration and resistance to thrombosis. Also, its dysfunction is associated with increased risk of atherosclerosis development. There are several potential links between chronic IBD-related inflammatory processes and the risk of cardiovascular disease, but insight into pathogenetic pathways remains unclear. We present the current concepts and review of adult and pediatric studies on the risk of CVD in IBD.

## Introduction

Cardiovascular diseases (CVD) are the major causes of mortality and morbidity worldwide, responsible for more than 4 million deaths in Europe each year [1]. The most common conditions are coronary heart disease and stroke strictly linked with classic CVD risk factors and health behaviors. The greatest challenge is continuous rise in the prevalence of obesity, arterial hypertension and diabetes. Individuals with inflammatory bowel diseases (IBD), ulcerative colitis (UC) or Crohn’s disease (CD) present with lower prevalence of classic CVD risk factors, like high BMI or lipid disturbances, compared to general population. Moreover, they typically enter the healthcare system at an earlier age, which potentially carries opportunities for effective early prophylaxis of CVD [2]. Therefore, “expected” lower cardiovascular morbidity and mortality are contradictory to “observed” higher prevalence; thus, IBD should be considered as a non-traditional risk factor for CVD in specific subsets of patients [3].

Moreover, the overall risk of serious cardiovascular events was related to IBD activity with highest risk during flares and periods of persistent activity [4].

There is increasing incidence of IBD in children and adults. The highest pediatric rates are in Europe with 23/100,000 persons per years [5], but the prevalence in newly industrialized countries in Asia, Middle East and Africa is increasing as well [6].

This “paradox” which may be also observed in other chronic inflammatory diseases such as rheumatoid arthritis or systemic lupus erythematous [7–9] hypothetically is linked with negative impact of global inflammation on arterial stiffening [10].

Recent findings have deeply changed the current view of coronary heart disease, going beyond the simplistic model of atherosclerosis as a passive process involving cholesterol build-up in the subintimal space of the arteries until their final occlusion and/or thrombosis and instead focusing on the key roles of inflammation and the immune system in plaque formation and destabilization [11].

Thrombosis in IBD is caused by the interaction of many hereditary and acquired risk factors with close link with inflammation. Higher levels of coagulation factors frequently occur in IBD which may predispose to arterial thromboembolic events [12]. The prevalence of venous thromboembolism (VTE) varies between 1 and 7% among patients with IBD. This is at least 3 times higher than that in the general population, and the absolute risk significantly increases during hospitalization, active disease and surgery [13].

There are several potential links between chronic IBD-related inflammatory processes, atherosclerosis, thrombosis and the overall risk of CVD; nevertheless, there is still lack of the clear insight into pathogenetic pathways. In this review, we present the current concepts and review of adult and pediatric studies on the risk of CVD in IBD.

### Possible links between IBD and CVD

**Fig. 1:**
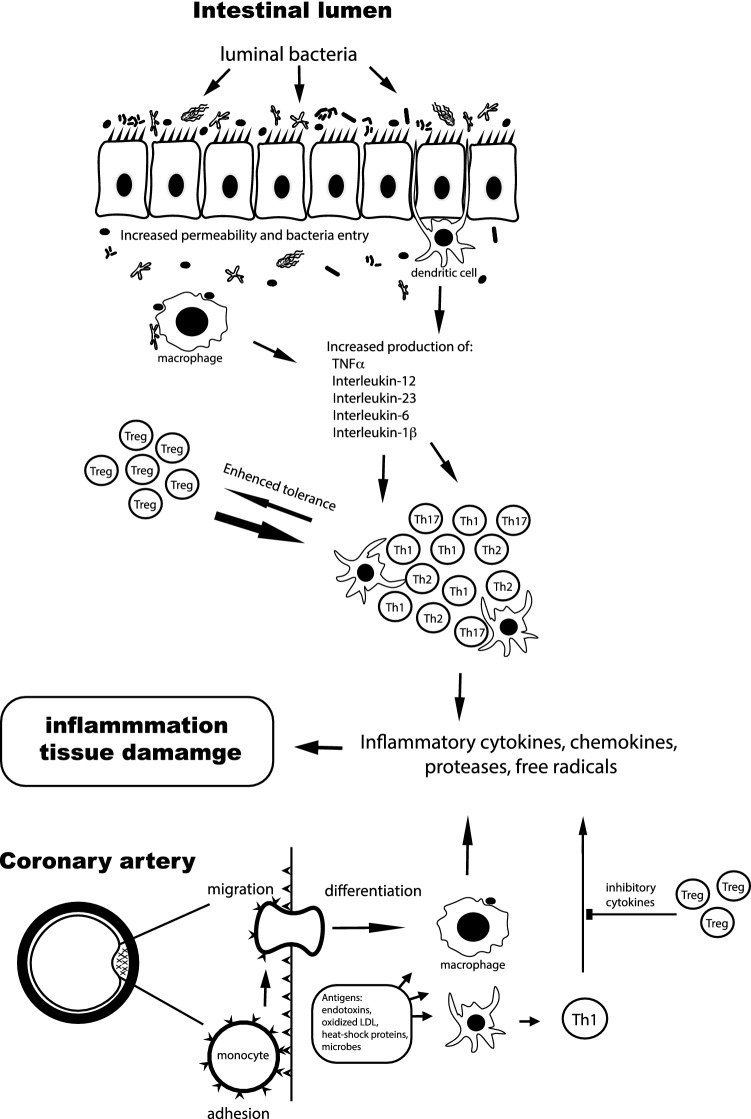
Potential pathogenesis of atherosclerosis and inflammatory processes in IBD. In intestinal inflammatory diseases, several pathways lead to increased bacterial exposure, including disruption of the mucous layer, dysregulation of epithelial tight junctions, increased intestinal permeability, and increased bacterial adherence to epithelial cells. In IBD, innate cells produce proinflammatory cytokines: TNF-α, interleukin-1β, interleukin-6, interleukin-12, interleukin- 23, and chemokines. There is also an increase in CD4+ T cell levels, especially in proinflammatory T-cell subgroups, which secrete proinflammatory cytokines and chemokines. Increased production of chemokines results in recruitment of additional leukocytes, which leads to cycle of inflammation. Monocytes recruited through the activated endothelium differentiate into macrophages. Several endogenous and microbial molecules can ligate pattern-recognition receptors (toll-like receptors) on these cells, inducing activation which results in release of inflammatory cytokines, chemokines, oxygen and nitrogen radicals, and other inflammatory molecules. This leads to inflammation and tissue damage. Antigens presented by macrophages and dendritic cells (antigen-presenting cells) trigger the activation of antigen-specific T cells in the artery. Most of the activated T cells produce Th1 cytokines which activate macrophages and vascular cells, leading to inflammation. Regulatory T cells modulate the process by secreting anti-inflammatory cytokines (such as interleukin-10 and transforming growth factor b). Adopted from Abraham and Hannson [16, 17]

We do not have clear insight into pathogenesis of IBD. Current concepts point at interplay among genetic susceptibility, abnormal mucosal immune response, defective epithelium and gut microbiome alterations [14, 15]. Roughly, gut flora invades defective intestinal mucosa and induces hyperactive immune response in a genetically predisposed individual. It results in dysregulation of inflammatory response involving TNF-α, INF-γ, IL-1β and cytokines of the IL-23-T_H17_ pathway [16] (Fig. 1).

Innate and adaptive immune mechanisms are involved in initiation and progression of atherosclerosis. Many types of inflammatory cells, i.e., macrophages, T and B lymphocyte, dendritic cells, are involved in plaque formation (Fig. 1) [17]. Thus, atherosclerosis formation may develop in two ways: direct way that takes place straight in the vessel and indirect one associated with cytokines released from non-vascular sites accelerating atherosclerosis [18].

Endothelium is a single layer of epithelium comprising inner surface of cardiovascular system. Its main role is to maintain a vascular homeostasis, and its dysfunction is deeply involved in CVD. Endothelium produces a number of mediators that regulate vascular homeostasis, i.e., nitric oxide (NO), prostacyclin, endothelin, von Willebrand factor and cellular adhesion molecules. It is responsible for blood coagulation, substance flow across vascular wall as well as leukocyte trafficking. Endothelium dysfunction is a state when the balance between vasodilating and vasoconstricting factors is disturbed, which leads to increased expression of cellular adhesion molecules, barrier malfunction with increased leukocyte diapedesis, elevated smooth muscle tone due to decreased vasodilatory substance production and increased vasoconstricting substances production [19, 20].

Chronic inflammation leads to structural and functional changes of the endothelium. During atherosclerotic plaque formation, T_H_1 response and INF-γ production lead to increase of TNF-α, Il-1, Il-6 and CRP; as a consequence, additional inflammatory and cytotoxic molecules are produced.

Long-term IL-6 and CRP levels are associated with CVD risk about as strongly as are some major established risk factors, highlighting their potential relevance as pathways to CVD [21, 22].

Human intestinal mucosal microvascular endothelial cells (HI-MECs) that upregulate adhesion molecules: ICAM-1, VCAM-1 and E-selectin were studied in vitro. It turned out that specimens from UC and CD patients exposed to proinflammatory stimuli have demonstrated higher rate of leukocyte adhesion than in controls [23]. Expression of another molecule selectively expressed in gut and recruiting lymphocytes—mucosal addressin cell adhesion molecule-1 (MAdCAM-1)—has also been elevated in UC and CD derived endothelium compared to healthy subjects [21]. Homocysteine plays its role in microvascular inflammation through endothelial activation of VCAM-1 expression, which leads to increased T cells and monocytes adherence [24].

The most potent vasodilating molecule, nitric oxide (NO) derives from L-arginin catalyzed by endothelial NO synthase. Patients with IBD exhibit increased expression of arginase, enzyme competing for l-arginine. TNF-alpha increases arginase activity leading to the decrease in NO production [25]. Circulating endothelial progenitor cells (EPCs) are the markers of endothelial repair. In patients with endothelial dysfunction, decreased production, impaired mobilization and increased apoptosis of these cells are observed. Garolla et al. investigated EPC in patients with IBD. The study has shown that EPC level was lower in UC and CD patients than in healthy controls [26]. Angiogenesis plays its role in gut inflammation maintenance mainly through CD40-CD40 ligand pathway leading to the secretion of proangiogenic cytokines [27].

The gut itself may have an impact on atherogenesis in IBD through its microbiota. Microbial products are released from the inflamed mucosa into the circulation through a leaky intestinal mucosal barrier. Translocation of microbial lipopolysaccharides (LPS) and other endotoxins induce expression of proinflammatory cytokines, which contribute to endothelial damage, atherosclerosis and cardiovascular events [26, 27]. There is possible role of Toll-like receptors in signaling pathways initiated by LPS. Toll-like receptors (TLRs) serve as the hub of immune responses to microbes in the gut in inflammatory bowel disease (IBD) pathogenesis. The abnormal TLR signaling may trigger disease-related inflammation. TLRs are key sensors in the gut to recognize abnormal intestinal microbes to induce immune response and inflammatory disease [28].

Increased expression of TLRs signaling pathways in monocytes was observed in active IBD, and TLR signaling pathways may play a role in atherosclerotic plaque activation [29].

Activation of TLRs promotes accumulation of foam cells in the aorta and migration of vascular smooth muscle cells to intima media.

### IBD and arterial stiffness

Increased carotid intima-media (cIMT) thickness and arterial stiffness are measurable markers of subclinical organ damage and independent cardiovascular risk factors. Systemic inflammation is an emerging causal factor for arterial stiffening in IBD accelerating smooth muscle hyperplasia and vascular fibrosis [30]. Vascular disease may involve pathogenic events within the vessel wall including not only atherosclerosis, but also arteriosclerosis, which are often used interchangeably, but they are different entities. Arteriosclerosis means the thickening and hardening of the walls of the arteries (within intima media), occurring typically in old age, while atherosclerosis is a disease of the arteries characterized by the deposition of fatty material on their inner walls [31].

So, arteriosclerosis differs from atherosclerosis in terms of site deposition (media vs. intima), form of deposition (diffuse vs. patchy) and clinical consequences (effects on cardiac function vs. focal ischemia) [31].

Microvascular alterations occur in affected but not unaffected intestinal wall [32, 33]. While normal intestinal microvessels vasodilate in response to acetylcholine using NO-dependent mechanisms, the intestinal endothelium in patients with IBD demonstrates several abnormal responses including the decreased responsiveness of microvessels to acetylcholine and an excessive dependence upon cyclo-oxygenase to maintain their vascular tone [34] Moreover, two angiographic studies of IBD patients have revealed an increased intestinal microvascular stenosis, abnormal vasa recta and diminished blood flow to the intestine [35, 36].

Finally, abnormal activation of the CD40/CD40L system has also been reported in IBD. This key regulator of immune reactivity was found to be elevated related to platelet activation that could be induced by passing the platelets through an inflamed microcirculation [37].

### Treatment of CVD in IBD

Disease activity may have an independent impact on the risk of acute CV events in patients with IBD. These findings may highlight new potential benefits of optimizing anti-inflammatory treatment in patients with persisting clinical activity **[**38].

Treatment of IBD is currently based on 5-ASA, corticosteroids, thiopurines and biological treatment. In a Danish population-based cohort of 4.6 million people, Rungoe et al. examined 28,833 Danish patients diagnosed with IBD from 1997 to 2009 and found that those who received 5-ASA had a decreased risk of ischemic heart disease compared to patients who have never received 5-ASA and the overall cardiovascular risk incidence compared to non-users. This cardioprotective effect was more stronger *n* patients who were treated with oral corticosteroids. In the same study, the authors observed a trend toward lower risk of ischemic heart disease in patients treated with thiopurines and/or TNF-α antagonists [36].

Aortic pulse wave velocity (aPWV) is an independent cardiovascular risk factor and a marker of subclinical organ damage. Zanoli et al. performed a subgroup analyses to test whether inflammation is associated with aortic stiffening. Analyzing data provided for 4 cohorts in 3 countries (151 participants with UC, 159 with CD and 227 control patients), authors have proved that the increased aPWV reported in patients with IBD is associated with inflammation. Compared with controls, aPWV was increased in patients with CD (mean difference 0.78 *z* score; 95% confidence interval, 0.56–1.00 *z* score [*P *< 0.001]) and UC (mean difference 0.75 *z*score; 95% confidence interval, 0.52–0.97 *z* score [*P *< 0.001]). Moreover, in their study aPWV was associated with disease duration (years, β = 0.05 *z* score; 95% confidence interval, 0.02–0.08 *z* score [*P *< 0.001]) and white blood cell count (billion cells/L, β = 0.07 *z* score; 95% confidence interval, 0.02–0.11 *z* score [*P *= 0.002]) but not with markers of acute inflammation (C-reactive protein and erythrocyte sedimentation rate), cardiovascular risk factors and therapy [39].

Positive effect of long-term anti-TNF-α therapy on aortic stiffness and cIMT progression was also shown in patients with rheumatoid arthritis and inflammatory arthropathies [40, 41].

Based on other models of chronic inflammation, for patients with a newly diagnosed vascular event, current standards of therapy—including warfarin, heparin, aspirin, thrombolysis, surgical thrombectomy and arterial stents—are indicated [42, 43]. The new generation of anticoagulants, including melagatran/ximelagatran and fondaparinux, are currently under evaluation for use in IBD patients with thrombotic events [44].

## Data from adults

Data from adults present contradictory data on association between IBD and cardiovascular diseases [45, 46].

In 2017, Feng et al. conducted a meta-analysis including 10 cohort studies investigating risk of ischemic heart disease in IBD. Investigators found out moderately increased risk of ischemic heart disease in IBD cohort (adjusted RR 1244). Sub-analysis revealed female, young age (< 50 years), short duration of follow-up (< 5 years) related to increased IHD risk [43]. In French National Hospital Discharge Database comprising 210 162 patients (97 708 CD) conducted in 2008–2013, 5554 patients experienced acute arterial event, and 24.8% of them had at least one of classic cardiovascular risk factor. Hypertension was the most prevalent (13%) one. The risk for acute arterial event was elevated in IBD patients aged from 15 to 34 years, and it was associated with disease activity in both UC and CD. In patients with no cardiovascular risk factors, acute arterial event risk was increased only in CD (SIR 1.26; 95 CI 1.19 to 1.34). No such a correlation was observed in UC (SIR 096 CI 0.92–1.01) [47]. Meta-analysis of observational studies (33 studies enrolled 72 205 IBD and 891 840 controls) revealed increased risk of thromboembolic events (TE) in IBD compared to controls by 96% RR 1.96. The risk of TE was comparable in CD and UC patients. The risk was higher in general IBD cohorts than in hospitalized patients. When considering each single thromboembolic event IBD population had increased risk of deep venous thrombosis, pulmonary embolism and mesenteric ischemia. On the contrary, the risk of stroke, arterial thromboembolism or peripheral artery disease was not increased comparing IBD and general population. The cardiovascular mortality rate was not elevated in IBD [48].

Danish study demonstrated VTE risk in IBD twice as high as in general population, slightly higher in CD than in UC. The risk was highest in the age group 0–20 years, and it lowered with increased age. However, the absolute risk increased with age. The VTE risk was still increased in the absence of provoking factor, i.e., surgery, fracture, malignancy [49]. Recently published population-based study from Denmark compared the occurrence of traditional and non-traditional cardiovascular risk factors between IBD patients and general population. The study included 108,789 subjects. Among them, 1203 had IBD diagnosis; 347 and 856 with CD and UC, respectively. Cardiovascular disease was more frequently diagnosed in IBD population than in general population: 13.2% and 10.9%, respectively, though the classic cardiovascular risk factors prevalence was lower. Patients with IBD had lower levels of total and LDL-cholesterol levels and higher hsCRP and fibrinogen than general population. The study confirms that higher CVD prevalence in IBD than in general population may be due to chronic inflammation [50].

The first population-based report on the incidence of venous thrombosis (VTE) and pulmonary embolism among IBD patients was from the UMIBDED study, which demonstrated that they have a threefold increased risk of developing deep VTE or PE [51]. Another study by Bernstein, which was based on UMIBDED, reported that the risk for coronary artery disease was increased with an incidence rate ratio (IRR) of 1.26 (95% CI 1.11–1.44) in both males and females with both CD and UC [52]. At the University of Miami, a four-year longitudinal cohort study of 356 persons with IBD and matched controls was performed. The unadjusted hazard ratio (HR) for developing coronary artery disease in the IBD group was 2.85 (95% CI 1.82–4.46). Despite the increased risk, people with IBD have significantly lower rates of selected traditional coronary artery disease risk factors (hypertension, diabetes, dyslipidemia and obesity; *P* < 0.01 for all). Adjusting for these factors, the HR for developing coronary artery disease between groups was 4.08 (95% CI 2.49–6.70) [3].

In meta-analysis of six studies, IBD was associated with a modest increase in the risk of coronary artery disease (odds ratio [OR] 1.19; 95% CI 1.08–1.31), both in CD an UC. This risk increase was seen primarily in women (four studies; OR 1.26; 95% CI 1.18–1.35), with no significant risk increase for men [53].

What factors may determine the increased risk of major CVD events if IBD is associated with lower rates of traditional vascular risk factors? Evidence from PROG-IMT Collaboration meta-analysis demonstrated a direct link between several inflammatory biomarkers, such as C-reactive protein, fibrinogen, leukocyte count with arterial stiffness and carotid intima media thickness (valid markers of premature atherosclerosis) [54]. Severe acute infections have also been associated with long-term major CVD events, potentially by inflammation-induced atherosclerotic plaque [55].

A French study to identify risk factors of acute arterial events in patients with IBD including 30 subjects with subsequent occurrence of acute arterial events (acute coronary syndrome or ischemic stroke) demonstrated that clinical disease activity and the persistence of systemic inflammation, diabetes, dyslipidemia and hypertension were significantly associated with an increased risk of acute arterial events (in univariate analysis). Neither protective nor aggravating effects associated with treatment exposure were identified. In multivariate analysis, the presence of diabetes (Odds ratio (OR) 14.5, 95% confidence interval (CI) 1.1 ± 184.7) and clinical disease activity (OR 10.4, 95% CI 2.1 ± 49.9) remained significantly associated with the risk of acute arterial event [38].

Grainge et al. performed the prospective study to quantify the risk of venous thromboembolism (VT) during different activity phases of IBD. Overall, IBD patients had a higher risk of VT than did controls (hazard ratio 3.4, 95% CI 2.7–4.3; *P* < 0.0001; absolute risk 2.6 per 1000 per person-years). At the time of a flare, this increase in risk was much more prominent (8.4, 5.5–12.8; *P* < 0.0001; 9.0 per 1000 person-years). This relative risk at the time of a flare was higher during non-hospitalized periods (15.8, 9.8–25.5; *P* < 0.0001; 6.4 per 1000 person-years) than during hospitalized periods (3.2, 1.7–6.3 *P* = 0.0006; 37.5 per 1000 person-years) [56].

In 2013, American gastroenterologist aimed to determine perceptions of VTE risks and self-reported practices regarding VTE prophylaxis in hospitalized IBD patients. A total of 135 eligible gastroenterologists responded to the survey. It turned out that there is significant variation in reported practices for VTE prophylaxis in IBD patients among gastroenterologists. Therefore, a more standardized approach to VTE prophylaxis should be implemented to improve health outcomes for IBD inpatients [57].

## Pediatric data

The pediatric data assessing cardiovascular risk are scarce.

Lurz et al. conducted a pilot study examining pulse wave velocity as a marker of arterial stiffness. Twenty-five children with IBD were involved—10 with UC and 15 with CD. There was male predominance in the study; mean disease duration was 2.8 years. At the time of the study, 2/3 of children were in clinical remission. None of them presented classic CVD risk factors unlike adults. Half of the patients received treatment with tumor necrosis factor (TNF). The results revealed that all children had PWV below 95 percentile. PWV in multivariate analysis was not linked to inflammatory markers, disease duration nor activity [58]. Aloi et al. investigated endothelial dysfunction through intima-media and flow-mediated dilation measurement. Twenty-seven children with CD, 25 with UC and 31 controls were included in the study. Thirty-seven percent enrolled CD patients were in clinical remission; mean PCDAI was 19.25 ± 15.54. In UC, majority of patients had chronic active disease; mean PUCAI was 35.15 ± 27.31. At the time of the study, 18 patients (35%) were on steroids, 10 (19%) on biological treatment, 19 (36.5%) received azathioprine and 5 (9%) methotrexate; 75% received mesalamine. Compared to controls in terms of classic cardiovascular risk factors, IBD had higher passive smoking exposure, higher diastolic blood pressure and lower BMI. Mean carotid intima-media complex was significantly higher in UC and CD than in controls. FMD was lower in both CD and UC patients. Patients with CD of severe course had worse findings than those with a mild course. There was no such a correlation in UC patients. The Italian study showed subclinical endothelial dysfunction in pediatric IBD cohort [59]. Aloi et al. continued an investigation of early atherosclerosis markers in IBD based on autopsy studies showing earlier atherosclerotic changes in abdominal aortic wall than in intima-media complex. Mean aortic intima-media thickness (aIMT) was significantly higher in IBD than in controls. Mean aIMT values were higher in both UC and CD than in controls. Univariate analysis found out significant association between aIMT and passive smoking, HDL values and inflammatory markers [60]. Trzeciak-Jedrzejczyk et al. assessed the risk of atherosclerosis in IBD studying adhesions molecules concentration (sICAM, sVCAM, sE-selectin) and lipid parameters. Forty children with IBD diagnosis and 21 children with no gastrointestinal disease were involved in the study. Twenty-six children had flare during the study. There was no statistically significant difference in average values of adhesion molecules between UC and CD nor to the control group. Patients with CD in remission had lower VCAM values than those with UC in remission. The level of TG was significantly higher in control group than in CD in remission. HDL cholesterol level was significantly higher in control group than in CD and UC patient with severely active disease. Investigators found no increased risk of atherosclerosis in children with IBD [61]. Winderman et al. assessed microvascular dysfunction that facilitates inflammation and thrombosis using plethysmography. Altered vascular flow was reported as reactive hyperemia index (HRI).

In the study, 16 IBD patients in clinical remission were compared to 16 healthy controls. The mean HRI values in IBD were lower than in healthy controls and within range associated with vascular disease [62].

Pac-Kożuchowska et al. performed the study comprising 30 children with first exacerbation of IBD to assess selected biomarkers of atherosclerosis in children with IBD. No significant differences were found in biomarkers of atherosclerosis in children with IBD compared to controls [63].

Although pediatric gastroenterologists are aware of the potentially increased risk of VTE in children with IBD, VTE prophylaxis is being provided by only about 1/3 of physicians. The main reason cited for not providing prophylaxis is lack of published literature, which emphasizes the need for collaborative clinical trials and guideline development [64].

Nylund et al. evaluated the risk for VTE in children and adolescents with IBD using a large population database. Their retrospective cohort study including 68,394 patients demonstrated that hospitalized children and adolescents with IBD are at increased risk for VTE. Conservative methods of VTE prevention such as hydration, mobilization or pneumatic devices should therefore be considered in hospitalized patients with IBD [65].

A Finnish study population consisting of 3551 children and adolescents which evaluated BMI, insulin, lipid, C-reactive protein and blood pressure levels, socioeconomic position, dietary habits and physical activity demonstrated that low childhood HDL cholesterol levels are associated with subsequent IBD diagnosis and a genetic risk score associated with low HDL cholesterol levels predict later IBD. Since HDL called a “good” cholesterol is well established as protective factor against cardiovascular complications, its low levels in young IBD patients may suggest a risk of increased CVD even at an early age [66]. Homocysteine plays role in microvascular inflammation through endothelial dysfunction [67, 68].

Nakano et al. performed cross-sectional study including 43 patients with IBD (27 Crohn’s disease, 9 ulcerative colitis and 7 indeterminate colitis) and 46 control to study the link between total homocysteine (tHcy) and IBD in children. Plasma tHcy concentrations were higher in children with IBD than in control subjects, which may also suggest that these patients are at higher risk of CVD development even at an early age [69]. Lazzerini at all performed a systematic review of studies on incidence and characteristic of thromboembolism (TE) in children with IBD. Population studies suggest that there is an increased risk of TE in children with IBD compared to controls. TE occurred in children with IBD in all age ranges, mostly (82.8%) during active disease, and more frequently in children with UC (odds ratio [OR] 3.7, 95% confidence interval [CI] 1.8–7.6) [70].

Zitomersky et al. performed retrospective review of 532 children and young adults with IBD to characterize thromboembolism (TE) in these patients at a single tertiary care hospital. The analysis revealed that pediatric inpatients hospitalized with IBD with colonic involvement have increased risk of TE, including complications of pulmonary embolism, recurrence, persistence and indefinite long-term anticoagulation. Therapeutic anticoagulation in IBD patients with active colitis appears safe. Both inherited thrombophilias and acquired risk factors were identified in patients with IBD and TE [71]. Therefore, the risk stratification should be used and prophylactic anticoagulation in high-risk patients is recommended [72].

Barclay et al. describe the incidence and outcome of cerebral thromboembolic events (CTE) in pediatric IBD patients from a single center over 5 years and the relative proportion of stroke reported in the literature in patients with UC and CD before and after January 2000. The study included 154 new patients diagnosed with IBD (male 56%) (UC 30%, CD 64%, IBD unclassified [IBDU] 6%). Four cases of CTE occurred in this population over 5 years (2.6%). All patients had a risk factor for CTE. Fifteen case series were identified with 32 patients. There was a significant increase in the proportion strokes affecting patients with CD reported after January 2000 (*P* = 0.02) [73].

On the other hand, due to conflicting data, Dorfman et al. investigated the association of IBD with CVD risk factors at late adolescence in a cross-sectional population-based study which consisted of 1 144 213 Jewish Israeli adolescents. Significant risk factors for CVD were not present in adolescents with IBD [74].

The summary of studies concerning CVD in IBD is presented in Table 1.

**Table 1:** The summary of CVD in IBD studies

References	Patients	Method	Investigating factor	Results	Risk factors
*Adult studies*					
Feng [27]	N/A	Ischemic heart disease in IBD	Meta- analysis	Moderately increased risk of ischemic heart disease in IBD	
Kirchgesner [28]	*N* = 210 162 CD = 97 708 UC = 112,452	Acute arterial events	Cohort study	Overall increased risk of acute arterial event in IBD	Female, Young age < 50, short duration of follow-up < 5
Fumery [29]	N/A	Thromboembolic event	Meta- analysis	Increased risk of TE in CD and UC	Age under 50, IBD-related hospitalization
Zanoli [32]	N/A	Aortic pulse wave velocity (aPWV) as an independent cardiovascular risk factor and a marker of subclinical organ damage	Meta-analysis	aPWV was increased in both CD and UC compared to the controls	
Bernstein [33]	*N* = 41,005 CD = 21,340 UC = 19,665	population-based cohort study	Incidence of venous thromboembolism (VTE) in IBD patients -pulmonary embolism	IBD patients have a threefold increased risk of developing DVT or PE.	Disease duration White blood cell count
Bernstein [34]	*N* = 80,489 Age (mean): CD = 33 UC = 39	population-based cohort study	Risk for coronary artery disease	IBD is associated with an increased incidence of cardiovascular events	
Yarur [35]	*N* = 356	Cohort study	Incidence of coronary artery disease (CAD) in IBD patients	An increased incidence of CAD events was noted in IBD patients despite having a lower burden of traditional risk factors.	
Singh [36]	N/A	Meta-analysis	The risk of arterial thromboembolic events: cerebrovascular accidents (CVA) ischemic heart disease (IHD).	IBD is associated with a modest increase in the risk of cardiovascular morbidity (from CVA and IHD)	Sex—female
Grainge [40]	*N* = 13,756	Prospective cohort study	Risk of VTE during different activity of IBD	The risk of VT was higher in IBD than in control The increase in risk was more prominent during the flare	Females Young age for CVA
Le Gall [39]	*N* = 3539 Age (mean): 42 y.o	Cohort Study	Occurrence of acute arterial events (acute coronary syndrome or ischemic stroke)—AAE	Active disease may increase the risk of AAE	Active disease Typical risk factors
*Pediatric studies*					
Aloi [43]	*N* = 52 CD = 27 UC = 25 Age (mean): 15.2 y.o	Endothelial dysfunction Flow-mediated dilation (FMD)	Cohort study	Higher mean carotid intima-media complex in IBD than in controls Lower FMD in IBD	
Lurz [42]	*N* = 25 Age (mean): 14.1 y.o	Cohort study (pilot study)	Not increased arterial stiffness in pediatric IBD		
Trzeciak-Jedrzejczyk [45]	*N* = 40	Adhesions molecules concentration and lipid parameters as risk factors of atherosclerosis	Cohort study	No statistically significant difference in average values of adhesion molecules between IBD and control group Higher TG level in controls than in CD in remission—higher HDL level in controls than in active IBD	
Winderman [46]	*N* = 16	Microvascular dysfunction	Cohort study	Lower hyperemia index	None
Pac-Kożuchowska [47]	*N* = 30 CD = 16 UC = 14 Age (mean): 13.2 y.o	To assess selected biomarkers of atherosclerosis in children with IBD	Cohort study	No significant differences were found in biomarkers of atherosclerosis in children with IBD compared to controls	None
Nylund [49]	CD = 61,076 UC = 7318 Age (range): 5–20 y.o	Risk of VTE in children and adolescence with IBD	Retrospective cohort study	Increased risk of VTE in IBD compared to controls	
Lazzerini [54]	N/A	Incidence of thromboembolism (TE) in children with IBD	Systemic review	An increased risk of TE in children with IBD compared to controls	
Zitomersky [55]	N/A	Retrospective review	Risk of thromboembolism (TE) in children and young adults with IBD	Increased risk of TE in young IBD patients	Active disease UC
Barclay [57]	*N* = 154 CD = 98 UC = 56	Retrospective cohort study	Incidence and outcome of CTE in pediatric IBD patients	2.6% of cases over 5 years	Colonic involvement Inherited thrombophilia

## Summary

Active disease or flare was reported as factors contributing to increase this risk in IBD patients, but not all studies have confirmed that observation. Nonetheless, it is important to know either pediatric IBD patients have higher risk of CVD and what are the risk factors in order to introduce potential prevention or treatment at an early stage [75] Especially, the overwhelming majority of the evidence points to increased risks for potentially life-threatening vascular disease in adults with IBD.

In summary, over the past years, a growing number of studies have indicated that IBD patients have an increased risk of developing CVD, and the reason is multifactorial. IBD, as a chronic inflammatory disease, may enhance atherosclerosis progression although clear pathological links were not established as yet. Moreover, higher levels of coagulation factors frequently occur in IBD and lead to thromboembolic events. Finally, the gut itself may have an impact on atherogenesis during IBD through microbiota alterations.

The problem of CVD risk in pediatric IBD is still underestimate and more studies are needed, although some data have demonstrated an increased CVD risk in children with IBD.
